# Fuzzy Divisive Hierarchical Associative-Clustering Applied to Different Varieties of White Wines According to Their Multi-Elemental Profiles

**DOI:** 10.3390/molecules25214955

**Published:** 2020-10-26

**Authors:** Ioana Feher, Dana Alina Magdas, Cezara Voica, Gabriela Cristea, Costel Sârbu

**Affiliations:** 1 National Institute for Research and Development of Isotopic and Molecular Technologies, 67-103 Donath, 400293 Cluj-Napoca, Romania; cezara.voica@itim-cj.ro (C.V.); gabriela.cristea@itim-cj.ro (G.C.); 2 Faculty of Chemistry and Chemical Engineering, Babeş-Bolyai University, 11 Arany Janos, 400028 Cluj-Napoca, Romania; csarbu@chem.ubbcluj.ro

**Keywords:** fuzzy associative-clustering, white wines, elemental profile, ICP-MS analysis

## Abstract

Wine data are usually characterized by high variability, in terms of compounds and concentration ranges. Chemometric methods can be efficiently used to extract and exploit the meaningful information contained in such data. Therefore, the fuzzy divisive hierarchical associative-clustering (FDHAC) method was efficiently applied in this study, for the classification of several varieties of Romanian white wines, using the elemental profile (concentrations of 30 elements analyzed by ICP-MS). The investigated wines were produced in four different geographical areas of Romania (Transylvania, Moldova, Muntenia and Oltenia). The FDHAC algorithm provided not only a fuzzy partition of the investigated white wines, but also a fuzzy partition of considered characteristics. Furthermore, this method is unique because it allows a 3D bi-plot representation of membership degrees corresponding to wine samples and elements. In this way, it was possible to identify the most specific elements (in terms of highest, smallest or intermediate concentration values) to each fuzzy partition (group) of wine samples. The chemical elements that appeared to be more powerful for the differentiation of the wines produced in different Romanian areas were: K, Rb, P, Ca, B, Na.

## 1. Introduction

Wine represents one of the most consumed beverages in the world and therefore has a large interest from an economic and social point of view. Wine quality is directly related to geographical origin, grape variety and technological processes. The wide variety of products results from different grape cultivars, vintages, geographic origins or winemaking techniques [1,2]. The geographic origin, grape variety and vintage of a specific wine are determined mainly by economic factors, and in turn, these factors determine the quality and price of wines from different regions, as well as the issue of trademark and consumer protection [1,2,3]. Wine composition is given by several factors like grape variety, soil influences and vinicultures practices (fertilizer or pesticide treatments), climate, and winemaking processes (yeast culture, aging, storage, quality and hygiene of vinery facilities) [4]. During the last years, many studies have focused on food and beverage fingerprinting and authentication, and one of the most important criteria followed is the recognition of the geographical origin of a certain product [5]. In this regard, an effective approach is determining the association between the matrix characteristics, obtained using different analytical methods, and multivariate statistical methods.

Wine is considered to be a complex matrix, having a wide range of organic and inorganic compounds [6], such as water, alcohols (ethanol and methanol), glycerin, sugars (glucose, fructose), organic (tartaric, malic, citric, lactic, acetic acid) and volatile acids [7], flavor compounds (esters, aldehydes, terpenes) [8,9], phenolic compounds (anthocyanins, tannins) [10], vitamins [11], minerals (anions and cations), and amino acids [12,13]. Wine quality evaluation and detection of fraudulent practices can be assessed through rigorous controls, which evaluate factors such as: geographical origin, grape varieties, vintages and oenological practices [14]. In Romania, the maximum acceptable limits for some elements in wine are set by Law 164/2015, which comprises the following values: As 0.2 mg·L^−1^, Cd 0.01 mg·L^−1^, Pb 0.2 mg·L^−^, Na 60 mg·L^−1^, Zn 5 mg·L^−1^, B 80 mg·L^−1^, Br 1 mg·L^−1^ and F 1 mg·L^−1^ [15].

The mineral composition of wine is mainly influenced by the soil composition of the vineyard, agricultural practices (i.e., fertilizers, pesticide) and the environment (pollution). Besides this, an important influence is the capacity of the grape variety to absorb minerals from the soil and also during the various steps of winemaking practices from grape to the final product [14,16,17]. Climatic changes may only affect the fungicide treatments, especially the level of Cu in grapes [18]. The elemental profile of wine is very often employed in the assessment of its geographical origin, as well as to determine its quality and safety, in terms of the maximum residue limits established by control authorities.

There are many reported studies dealing with the assessment of wine authenticity according to their mineral profiles using distinct techniques like: flame atomic absorption spectroscopy (FAAS), graphite furnace atomic absorption spectroscopy (GFAAS), voltammetry, capillary electrophoresis, inductively coupled plasma mass spectrometry (ICP-MS) and inductively coupled plasma optical emission spectrometry (ICP-OES), assisted in many cases by chemometrics [19,20,21]. From all these, ICP techniques are the most extensively employed for geographical traceability of food products, because of the proven low detection limits, multi element determinations, and wide dynamic ranges. In order to assure superior valorization of the results, the association with different chemometric techniques is often applied for different food matrices, such as: honey [22], juices [23], wines [24], dairy products [25,26] or vegetables [27,28].

In this study, the fuzzy divisive hierarchical associative-clustering method, which gives an excellent overview to associate each fuzzy partition of samples to a fuzzy set of characteristics (specific chemical elements), was successfully applied for modelling 65 Romanian white wines according to their elemental profile. The novelty of this study is represented by the partitioning of white wines and their association with different chemical elements with high, moderate and low concentrations. The obtained results clearly demonstrated the efficiency and information power offered by the advanced fuzzy clustering method in wine characterization and authentication.

## 2. Results

### 2.1. Descriptive Statistics

The elemental data used in this study were obtained for 65 Romanian white wines (Table S1). It is easy to observe the important differences among the elemental concentrations of the investigated elements (Table 1). The highest concentrations (expressed in μg·L^−1^), in decreasing order, were obtained for K (238898.5), P, Mg, Ca, Na, and Rb (1237.1), moderate concentrations were identified for Mn (676.8), Sr, Zn, Al, Ba, and Cu (81.5), while the lowest concentrations corresponded to Pb (12.4), Li, Sc, Ce, Cs, Pd, Bi, Co, As, Be, Ga, U, Tl, Ag, Au, Sb, and In (0.2).

### 2.2. Fuzzy Divisive Hierarchical Associative-Clustering

For comparison of similarities and differences among white wine sample partitions, the DOMs (degrees of memberships) corresponding to all fuzzy partitions for both the samples and characteristics (elements concentrations) were analyzed. The results obtained by applying the fuzzy divisive hierarchical associative-clustering method using the elemental data are presented in Table 2.

By analyzing the fuzzy partitions at each level (partition history/hierarchy) along with the elemental considered data, the following observations may be taken. At the first partition level the white wines (65 samples) are separated into two fuzzy partitions A1 and A2, respectively. The degrees of membership (DOMs) of the wines included in partition A1 are in the range 0.9836–0.7428 or 98.36–74.28%, and between 0.9998 and 0.6449 (99.98–64.49%) in the case of A2. Most of the wines assigned to partition A1 belong to the Sauvignon Blanc cultivar from different areas (4 from Moldova, 2 from Oltenia and 1 from Muntenia), except one sample (Italian Riesling) from Oltenia. Potassium is the single element in this group associated with a very high DOM (0.9990). The concentration of K is the highest for all seven wines and is quite different from the rest of investigated samples. Other authors found a link between the elemental composition of wines and soil, with K having the maximum concentration in analyzed soil samples, among investigated macroelements [29].

At the second level, only partition A2 is divided, resulting in partitions A21 and A22, with DOMs between 0.9867 and 0.5665 for A21 and from 0.9884 to 0.6115 for A22. The elements assigned to partition A21 according to their DOMs (0.9090–0.7406) are Mg, Ca and P. The concentrations of these elements are very high, but are comparable and close for the samples assigned to this group (the majority from Moldova); only the vintage and cultivar are different. The elements that were grouped in this partition are applied in different areas, suggesting similar agricultural practices, especially related to the use of fertilizers. The application of multiple element fertilizers (calcium, magnesium, iron, manganese, copper, zinc and boron) might affect both the yield of the wine grapes and the content of tannins, anthocyanins or total phenols [30].

On the contrary, the A22 partition includes only samples from Transylvania, Muntenia and Oltenia and all the elements are associated with very high DOMs (0.9996–0.9817). The sample group A221 contains a sample originated mainly from the Transylvania area and among associated variables are Sr, Li, Mn, Au and Ag. Our previously reported papers [25,26,27] found similar geographical markers for other food products (raw or processed) grown in Transylvania (milk, cheese, potatoes); a fact that is confirmed by our results. Moreover, the content of Au and Ag could reflect wine from Transylvania, due to the fact that there are some important gold mining areas there that contain important reserves of the above-mentioned elements, and, through natural leaching processes, these elements are spread on the surrounding area, ending up in the food products.

The partition A21 is divided to the final partition A211, A2121 and A2122. The majority of the samples assigned to A211 are from Moldova and the associated element is P, with a relatively high DOM (0.7405). A possible explanation in this regard is the agricultural practices that are undertaken, where phosphorus-based fertilizers are a key component for a healthy plant development.

The partition A2121 contains only two samples from two different areas (Moldova and Muntenia) and has only Mg as an associated element. The common features in this partition are represented by the year 2012, which can be linked by the similar meteorological conditions from the two geographical areas.

The partition A2122 includes samples from three different areas (Moldova, Oltenia and Transylvania) and Ca as a specific element (DOM = 0.7837). The elements associated to the group corresponding to the partition A222, with a very high DOM, are Na (0.9598) and B (0.9097), and include mainly samples from Muntenia and some samples from Oltenia and Transylvania. The partition A2211 contains only samples from Transylvania with various DOMs (0.8888–0.4492) and the element associated with a very high DOM (0.9422) is Rb. It seems that Rb, among other before-mentioned geographical markers, is very characteristic for the Transylvania area [28].

**Figure 1:**
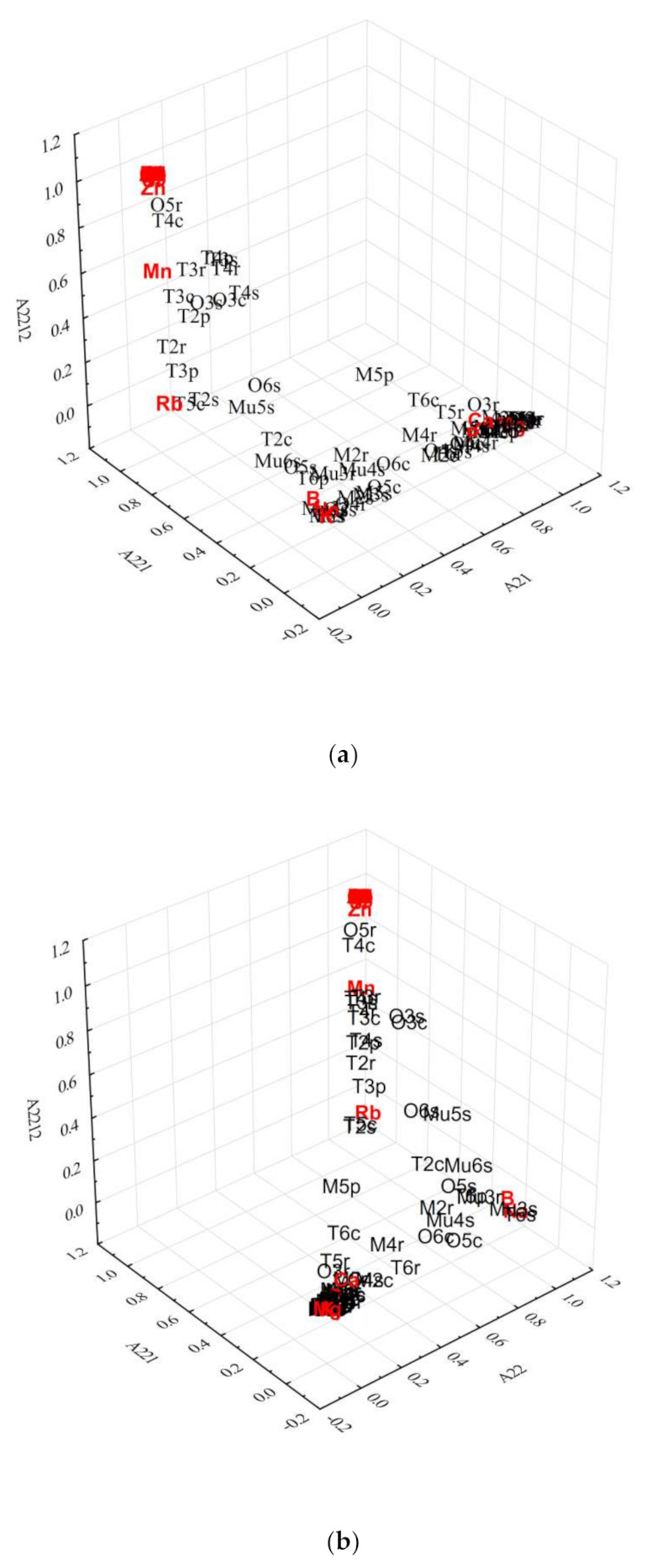
3D bi-plot of DOMs corresponding to fuzzy partitions (**a**) A21, A221 and A2212 and (**b**) A22, A221 and A2212.

The partition A2212 includes many samples from Transylvania and some from Oltenia, but with different DOMs (0.8945–0.2854), and it has many associated elements that have, in many cases, a low concentration. The partition A22121 is more or less similar to A2212, but the partition A22122 is more interesting because it includes only samples from Transylvania and the most characteristic elements are Zn (0.9364), Sr (0.8097) and Mn (0.4689). Sr is an acknowledged marker for geographical differentiation, while Mn content was proved by our previously reported studies [27] to be an important marker/discriminator for Transylvanian food products. The partition A221211 includes the majority of the samples corresponding to A22121 except two wines (1 from Transylvania and 1 from Oltenia assigned to A221212), and also the majority of elements with the lowest concentration, all with different DOMs. The elements assigned with relatively high DOMs to A221212 are Al, Ba and Cu. The last two partitions A2221 and A2222 contain samples from different areas, with very different DOMs; B being associated with partition A2221 and Na with A2222. The DOMs of these two elements are very high (0.9093 and 0.9591) and the majority of samples included in partition A2221 are from Muntenia. All of the above statements are very well supported by the 3D bi-plot of DOMs corresponding to different fuzzy partitions as is illustrated in Figure 1a,b.

## 3. Materials and Methods

### 3.1. Sample Collection

For this study, 65 Romanian white wine samples were collected from four geographical vineyards (Transylvania-20, Muntenia-8, Oltenia-18 and Moldova-19). The investigated cultivars were as follows: Sauvignon Blanc-23, Riesling-20, Chardonnay-13 and Pinot Gris-9. The wine sample distribution according to their vintages was: 2012-15 samples, 2013-13, 2014-15, 2015-11 and 2016-11.

### 3.2. ICP-MS Analysis

For sample dilution and preparation of standards, ultrapure deionized water (18 MΩ·cm^−1^) from a Milli-Q analytical reagent grade water purification system (Millipore) was employed. For wine digestion, nitric acid ultrapure grade (69% Merck) was used. The wine samples were prepared according to the following procedure [31]: 2.5 mL of ultra-pure nitric acid were added to 2.5 mL of wine in a Teflon receptacle, tightly closed. Six devices were inserted into stainless steel cylinders placed between two flanges, for pressure resistance. The whole system was put in an oven at 200 °C for 12 h. A colorless solution resulted and ultra-pure water was made up to 50 mL. Thus, the wine sample was diluted 1:20 *v*/*v* and directly analyzed by ICP-MS.

Element determinations were carried out with a PerkinElmer ELAN DRC (e) ICP-MS apparatus, equipped with a Meinhart nebulizer and silica cyclonic spray chamber and continuous nebulization. A smart tune solution (Perkin Elmer Pure Plus), containing 10 μg·L^−1^ each of Ba, Be, Ce, Co, In, Mg, Pb, Rh, U in a matrix of 1% HNO_3_ was used for ICP-MS instrument optimization in order to achieve Ba++/Ba+ and CeO/Ce < 3%. The instrumental conditions were: Nebulizer Gas flow rates: 0.86 L/min –Auxiliary Gas Flow: 1.2 L/min–Plasma Gas Flow: 15 L/min–Lens Voltage: 7.25 V.–ICP RF Power: 1100 W–CeO/Ce = 0.031–Ba++/Ba+ = 0.016. A primary analysis was performed using a semiquantitative method available with Perkin-Elmer ICP-MS instrumentation (Total Quant method). A solution of 10 µg∙L^−1^ Mg, Cu, Cd, In, Ba, Ce, Pb, U in 1% HNO_3_ (Perkin Elmer Atomic Spectroscopy Standard–Setup/Stab/Masscal Solution) was used as an external standard. The explored mass domains were 6 < *m*/*z* < 14; 22 < *m*/*z* < 37; 40 < *m*/*z* < 238. The background signal (blank) was determined with an ultra-pure water sample.

For the quantitative method, calibration standards for multi-element determinations were prepared by successive dilution of four high purity ICP, Multi-Element Calibration Standards (Perkin Elmer Life and Analytical Sciences): Standard 3 (10 µg∙mL^−1^ Al, As, Ba, Be, Bi, Ca, Cd, Co, Cr, Cs, Cu, Fe, Ga, In, K, Li, Mg, Mn, Ni, Pb, Rb, Se, Na, Ag, Sr, Tl, V, U, Zn); Standard 2 (10 µg∙mL^−1^ Ce, Sc), Standard 4 (10 µg∙mL^−1^ Au, Pd, Sb) and Standard 5 (10 µg∙mL^−1^ B and P). For each sample analysis, three replicates were performed. The precision, expressed as relative standard deviation, was under 5%. Accuracy was expressed by recovery tests carried out for a wine sample spiked with a 2.5 μg·L^−1^ standard solution. The % of recovery was in the range of 85–110%.

### 3.3. Fuzzy Clustering Methods

Fuzzy analysis and fuzzy logic represent useful and powerful tools in analytical chemistry and other scientific and technical fields [32,33,34,35]. Clustering and classification methods are useful since they allow meaningful generalizations to be made about large quantities of data sets by recognizing general patterns among them [36]. There are a lot of algorithms that aim to give principal results as hard clusters from a given data set, c-means algorithms being the most widely used. Hard c-means methods execute a sharp clustering, in which each object (sample) is either assigned to a cluster or not. The membership of objects to a specific cluster is assigned to values between 0 and 1. The application of a Fuzzy algorithm in a clustering approach causes this cluster membership to become a relative one and consequently an object can belong to several clusters at the same time, but with different degrees of membership (DOMs) between 0 and 1. The DOMs to which a given data point belongs to the different clusters, are computed from the distances of the data point to the cluster centers (prototypes). The closer a data point is to the center of a cluster, the higher is its degree of membership to this cluster.

Clustering methods can be split in two types, as follows: partitional clustering and hierarchical. Partitional clustering methods try to directly decompose a data set into a fixed number of disjoint clusters (semi-supervised), while the divisive hierarchical clustering algorithm is an unsupervised procedure and may be used to obtain the cluster structure of the data set when the number of clusters is unknown.

Using the generalized fuzzy c-means algorithm (GFCM) [37], one can determine a binary fuzzy partition {A1, A2} of the data set X. If this partition describes real clusters, it is denoted P^1^ = {A1, A2}. Using the GFCM algorithm for two subclusters (*n* = 2), one can determine a binary fuzzy partition for each Ai of P^1^. If this partition of Ai describes real clusters, these clusters will be attached to a new fuzzy partition, P^2^. Otherwise, Ai will remain undivided. The cluster Ai will be marked and will be allocated to the partition P^2^. The unmarked cluster members of P^2^ will follow the same procedure. The divisive procedure will stop when all the clusters of the current partition P^k^ are marked, that is there are no more real clusters. This procedure is a divisive one and gives the possibility of performing fuzzy hierarchy [38,39,40,41].

Considering now a fuzzy partition of the fuzzy set C of objects, and Q, a fuzzy partition of the fuzzy set D of characteristics (variables), the problem of the fuzzy divisive hierarchical associative-clustering is to determine the pair (P, Q) that optimizes a certain criterion function [33,34,35]. By starting with an initial partition P^0^ of C and an initial partition Q^0^ of D, a new partition P^1^ will be obtained. The pair (P^1^, Q^0^) allows the determination of a new partition Q^1^ for characteristics. The algorithm consists of producing a sequence (P^k^, Q^k^) of pairs of partitions, starting from the initial pair (P^0^, Q^0^), in the following steps:

(1) $$\left(\mathrm{Pk},\;\mathrm{Qk}\right)\;\to \;\left(\mathrm{Pk}+1,\;\mathrm{Qk}\right)$$

(2) $$\left(\mathrm{Pk}+1,\;\mathrm{Qk}\right)\;\to \;\left(\mathrm{Pk}+1,\;\mathrm{Qk}+1\right)$$

The rationality of the divisive hierarchical associative-clustering method essentially supposes the splitting of the sets X and Y in two subclasses. The obtained classes are further divided in two subclasses, and so on. The two hierarchies may be represented by the same tree, having a pair (C, D) in each node (level of partition), where C is a fuzzy set of objects and D is a fuzzy set of characteristics. As a first step, the fuzzy partitions of the classes C and D must be simultaneously determined (as a particular case, the binary fuzzy partitions), so that the two partitions should be highly correlated. With the GFCM algorithm, a fuzzy partition P = A1, …, An of the class C will be determined using the original characteristics. In order to classify the characteristics, the algorithm computes their values for the classes A_i_, i = 1, …, c. The value ${\bar{y}}_{i}^{k}$ of the characteristic *k* with respect to the class A_i_ is defined as:

(3) $${\bar{y}}_{i}^{k}=\overset{n}{\underset{j=1}{\sum }}\begin{array}{c} A_{i}\left(x^{j}\right)x_{k}^{j},\;i=1,\;\ldots .c;k=1,\;\ldots .d \end{array}$$

where A_i_(x^j^) is the membership degree of object x^j^.

## 4. Conclusions

In order to understand the distribution pattern of chemical elements in various Romanian white wine samples, collected from four geographical areas, fuzzy divisive hierarchical associative-clustering was successfully applied. The fuzzy partition hierarchy of samples and associated chemical elements allowed us to identify partitions (groups) of wine samples with more or less similar characteristics in terms of higher, smallest or intermediate values of concentration. In addition, the 3D bi-plot representation of DOMs corresponding to different fuzzy partitions offers the possibility of visualizing the relationship among samples and specific elements. Some elements appeared to be more specifically (markers) related to the geographical origin of wines (K, Rb, P, Ca, B, Na). For Transylvanian wines, some specific markers were highlighted, namely: Sr, Li, Ag, Au and Rb, while for wines originated from Moldova, P was the most representative characteristic. It was possible to determine the vintage in one case, 2012, through Mg content. Apart from the above-mentioned differentiations, the similarities in the agricultural practices were also noticed among the analyzed wine samples.

## Figures and Tables

**Table 1:** Descriptive statistics of the concentration (μg·L^−1^) of 30 elements, for investigated Romanian white wines (*n* = 65).

Element	Mean	Median	Minimum	Maximum	Range	Std.Dev.
Li	11.8	8.8	0.0	75.1	75.1	12.9
Be	0.6	0.0	0.0	5.9	5.9	1.0
B	4896.3	5015.2	1654.2	9120.4	7466.2	1703.1
Na	7352.3	5904.8	2429.6	34053.5	31623.9	5345.5
Mg	54434.8	53676.1	37390.4	92725.6	55335.2	10216.3
Al	188.2	49.1	0.0	1857.9	1857.9	337.8
P	78044.8	72164.4	9573.5	197524.9	187951.4	37460.6
K	238898.5	214348.2	123688.4	719534.1	595845.8	101572.9
Ca	39343.1	39865.4	20474.6	62776.9	42302.3	10256.6
Sc	5.2	5.0	1.7	11.1	9.3	1.9
Mn	676.8	612.1	281.6	1569.4	1287.8	297.7
Co	1.2	0.2	0.0	7.6	7.6	1.9
Cu	81.5	29.5	0.0	1020.0	1020.0	157.3
Zn	285.7	219.6	0.0	1116.9	1116.9	247.6
Ga	0.5	0.4	0.0	1.6	1.6	0.4
As	1.0	0.7	0.0	4.0	4.0	1.0
Rb	1237.1	1228.2	310.7	3025.5	2714.8	534.0
Sr	302.7	249.5	118.6	1035.9	917.3	184.8
Pd	4.1	3.6	0.0	14.4	14.4	3.5
Ag	0.3	0.2	0.0	1.8	1.8	0.4
In	0.2	0.1	0.0	1.2	1.2	0.2
Sb	0.2	0.2	0.0	0.5	0.5	0.1
Cs	4.2	3.8	0.7	10.2	9.5	2.1
Ba	84.5	76.2	20.5	220.2	199.7	40.4
Ce	4.6	0.9	0.0	94.0	94.0	13.0
Au	0.3	0.3	0.0	1.0	1.0	0.2
Tl	0.4	0.3	0.0	1.5	1.5	0.3
Pb	12.4	6.7	1.6	91.4	89.8	15.2
Bi	1.4	1.0	0.0	4.6	4.6	1.1
U	0.4	0.3	0.0	2.2	2.2	0.4

**Table 2:** The fuzzy divisive hierarchical associative-clustering partitions of wines and variables (wines and chemical elements are arranged in decreasing order of DOMs).

Fuzzy Partition Level	Divisive Fuzzy Partition History	Wines	DOMsRange of Wines	Associated Variables (Elements)	DOMs Range of Variable (Elements)
0	A	1, …, 65		1, …, 30	
1	A1	M2s, O2s, O4r, M6s, M3s, Mu2s, M5s	0.9836–0.7428	K	0.9990
	A2	T6c, T6r, T5r, T2c, Mu4s, T6p, O3r, O6c, M2s, O5s, M2p, Mu3r, Mu6s, T6s, O6c, Mu5s, T5p, M4r, O3c, O3s, Mu3s, M4r, O6s, O2r, Mu2r, O5c, T5c, T2p, T3p, T4c, M5p, T2s, M2r, O6r, T3r, M4p, T2r, T3s, M4s, T4r, T3c, O5r, M2r, T4s, T4p, M3s, M4c, M6r, M5r, M3r, O2c, M3p, M2c, O4s, Mu4r, O4c, M4s, T5s	0.9998–0.6449	B, Na, Rb, Mn, Sr, Zn, Al, Cu, Ba, Pb, Li, Sc, Ce, Pd, Cs, Bi, Co, As, Be, Ga, U, Tl, Ag, Au, Sb, In, Ca, Mg, P	1.0000–0.8069
2	A21	O2r, M4r, O6c, T5p, O6r, Mu2r, M2r, M2p, M4s, M2s, M4p, O3r, O2c, M3p, M3s, M4c, M6r, M3r, T5r, M5r, Mu4r, M4s, O4c, T6c, T5s, T6r, O4s, M4r, M2c, M5p	0.9867–0.5665	Mg, Ca, P	0.9090–0.7406
	A22	O3s, Mu5s, T6s, Mu6s, Mu5s, T3p, T2r, T4c, O5r, O3c, T3c, T5c, T3r, T2p, T6p, O5s, O6s, T2c, T2s, Mu3r, T4p, T3s, T4r, T4s, Mu4s, M2r, O5c, O6c	0.9884–0.6115	Mn, Rb, Sr, Zn, Al, Cu, Ba, Pb, Li, Sc, Ce, Pd, Cs, Bi, Co, As, Be, Ga, U, Tl, Ag, Au, Sb, In, B, Na	0.9996–0.9817
3	A211	O6r, M4s, M3p, M3s, M4p, M4r, M2s, T5p, M4c, Mu4r, M4s, T5s, O4c, O3r, M6r, M2c, T6r	0.9214–0.3494	P	0.7405
	A212	M2p, M2r, Mu2r, O2r, T5r, M3r, O6c, T6c, O4s, M5r, M4r, O2c, M5p	0.8803–0.3904	Mg, Ca	0.8604; 0.7837
4	A2121	M2p, Mu2r	0.8726–0.7183	Mg	0.8604
	A2122	T5r, O6c, M2r, O2r, T6c, O4s, M4r, O2c, M5r, M3r, M5p	0.7537–0.3608	Ca	0.7837
5	A221	T4c, T2r, O5r, T5c, T3c, T3p, T2p, T3r, T2s, T4p, T3s, T4r, O3s, T4s, O3c, O6s	0.9471–0.4844	Al, Cu, Ba, Pb, Sr, Li, Ce, Sc, Pd, Cs, Bi, As, Co, Be, U, Ga, Ag, Au, Tl, Sb, In, Zn, Mn, Rb	0.9994–0.9442
	A222	T6s, Mu3s, Mu3r, T6p, O5c, Mu6s, O5s, Mu4s, Mu5s, O6c, M2r, T2c	0.9830–0.4454	Na, B	0.9598; 0.9097
6	A2211	T5c, T2s, T3p, T2r, T2p	0.8888–0.4492	Rb	0.9422
	A2212	O5r, T4c, T4p, T3s, T4r, T3r, O3c O3s, T4s, T3c, O6s	0.8945–0.2854	Al, Ba, Pb, Ce, Li, Sc, Pd, Cs, Bi, As, Be, Ag, Co, Au, U, Ga, Tl, In, Sb, Cu, Sr, Zn, Mn	0.9989–0.5792
7	A22121	O5r, T4c, T3r, O3s, O3c, T3c, O6s	0.7895–0.2543	Al, Ce, Li, Sc, Pd, Cs, Bi, As, Be, Au, Ag, U, Ga, Tl, Co, Sb, In, Al, Ba, Cu	0.9981–0.7604
	A22122	T4p, T3s, T4r, T4s	0.6889–0.5368	Zn Sr Mn	0.9364–0.4689
8	A221211	T4c, T3r, O3s, O3c, O6s	0.7881–0.2496	Li, Pd, Cs, Sc, Bi, As, Be, Au, Ag, U, Ga, Tl, Co, Sb, In, Pb, Ce	0.9965–0.9734
	A221212	O5r, T3c	0.7766; 0.3596	Al, Ba, Cu	0.8573–0.7597
9	A2221	Mu3s, T6s, Mu6s, O5c, O5s, Mu4s, Mu5s, M2r, T2c	0.9204–0.4239	B	0.9093
	A2222	Mu3r, T6p, O6c	0.7292–0.5125	Na	0.9591

## Supplementary Materials

The following are available online at. Table S1: Multi-elemental concentrations (μg/L) measured in wine samples, collected from four different areas (T-Transylvania, Mu-Muntenia, O-Oltenia, M-Moldova).
