# Supplementary material for: Fuzzy Divisive Hierarchical Associative-Clustering Applied to Different Varieties of White Wines According to Their Multi-Elemental Profiles
Source: Molecules. 2020 Oct 26;25(21):4955. doi: 10.3390/molecules25214955 (PMC7662284; doi:10.3390/molecules25214955)
Supplement: Supplementary file 1 [file molecules-25-04955-s001.pdf]

Table S1. Multi-elemental concentrations (µg/L) measured in wine samples, collected from four different areas (T-Transylvania, Mu-Muntenia, O-Oltenia, M-Moldova)

Wine cultivar	Area	Li	Be	B	Na	Mg	Al	P	K	Ca	Sc	Mn	Co	Cu	Zn	Ga	Tl	Pb	Bi	U
Pinot Gris	T	1.52	0.02	3116.2	2688.56	41503.26	<0.001	26817.50	218138.90	47948.54	4.16	410.70	<0.001	89.52	52.76	0.26	0.66	34.64	3.50	0.42
Sauvignon Blanc	T	4.72	0.74	4814.74	2712.32	43190.68	46.62	16929.10	205354.30	48164.88	5.72	1328.06	0.10	21.98	138.36	0.26	0.36	34.46	2.28	0.42
Riesling Italian	T	1.52	0.02	3679.14	3354.38	50018.72	101.72	33176.86	155216.20	47663.90	4.80	364.02	1.02	19.60	987.66	0.64	0.80	75.96	4.46	0.62
Chardonnay	T	4.72	0.74	5358.76	4385.06	43788.00	121.50	63767.48	209403.70	46513.92	5.38	458.54	0.06	88.88	87.44	0.50	0.36	91.40	0.64	0.30
Sauvignon	T	17.52	0.74	4120.48	3727.82	39098.68	<0.001	9573.52	208172.80	47641.12	3.36	323.44	<0.001	40.32	200.96	0.24	<0.001	16.42	0.64	0.10
Chardonnay	T	2.68	<0.001	3791.26	4012.04	43738.22	<0.001	19646.74	148432.20	62776.86	3.64	484.16	0.10	515.58	358.94	0.04	0.18	14.74	1.90	0.10
Pinot Gris	T	0.64	<0.001	5676.12	2429.58	46072.16	<0.001	38147.30	174651.80	43291.96	3.12	695.72	<0.001	36.36	80.62	<0.001	0.42	8.76	2.18	0.20
Riesling Italian	T	5.00	<0.001	2102.52	2501.76	42327.50	<0.001	71032.06	147103.40	36416.52	4.76	508.78	<0.001	164.24	242.24	0.76	0.18	16.06	1.12	0.10
Riesling Italian	T	20.72	0.74	3440.60	4265.58	37390.42	<0.001	35244.50	195304.30	25539.90	6.48	470.10	<0.001	196.34	<0.001	0.10	<0.001	35.74	1.52	<0.001
Sauvignon	T	0.64	<0.001	3524.10	3355.02	38984.38	3.34	11236.88	211557.70	36143.36	6.20	366.12	<0.001	122.72	210.02	<0.001	0.06	21.62	2.74	0.20
Chardonnay	T	<0.001	0.74	2985.00	3954.02	53183.58	<0.001	40715.56	163629.60	49007.48	5.66	728.30	<0.001	41.70	97.04	0.04	0.30	10.34	0.54	0.20
Pinot Gris	T	2.38	2.20	3886.68	4164.82	42679.00	<0.001	28737.14	146606.9	32137.22	5.12	504.00	<0.001	170.20	242.48	1.04	0.28	10.22	3.78	0.62
Chardonnay	T	16.94	0.02	4137.18	14500.42	60006.36	20.12	69399.98	196549.20	31329.24	6.38	708.36	1.34	530.20	680.78	0.24	0.30	4.34	1.16	0.20
Riesling Italian	T	21.30	0.74	5921.92	3787.44	76610.16	205.16	114544.30	233413.20	36587.26	7.28	1312.36	3.08	24.44	230.48	1.00	0.54	16.14	0.16	0.30
Sauvignon Blanc	T	6.16	<0.001	8244.24	3873.78	50020.40	<0.001	77381.76	637693.40	30942.32	7.68	605.76	0.04	31.60	97.98	0.80	0.30	1.58	0.46	0.62
Sauvignon Blanc	T	19.84	<0.001	6303.74	6169.66	57062.84	<0.001	56432.96	199948.10	29750.52	5.96	357.46	0.48	26.14	322.00	<0.001	0.30	9.40	1.42	0.10
Pinot Gris	T	2.68	0.02	2894.36	18917.54	40783.30	76.86	64522.30	202312.00	40366.30	2.10	917.62	0.34	105.84	131.38	0.16	0.06	15.60	0.32	<0.001
Sauvignon	T	0.06	0.74	4461.64	9990.44	51250.08	<0.001	39924.56	220055.10	46149.60	2.36	894.12	<0.001	10.36	38.38	<0.001	<0.001	12.90	1.40	0.42
Riesling Italian	T	9.96	0.02	2248.00	9895.72	44644.48	<0.001	89298.28	204291.30	37736.86	1.82	365.56	0.32	1019.96	53.20	0.30	0.40	20.34	2.42	0.10
Chardonnay	T	2.68	1.48	3309.40	3849.04	48339.94	192.36	77406.76	209256.80	48301.52	2.46	310.74	0.02	381.90	169.02	0.70	0.06	16.40	0.02	0.30
Sauvignon Blanc	Mu	58.56	2.2	7244.08	5886.14	52898.00	285.58	197524.90	719534.10	62093.36	7.28	738.52	3.22	122.72	419.54	1.58	0.24	4.48	1.50	0.10
Riesling Italian	Mu	29.16	<0.001	5482.84	6873.10	92725.62	<0.001	72164.44	186730.00	35540.14	6.30	913.76	<0.001	29.52	91.80	<0.001	0.12	6.86	1.28	0.42
Riesling Italian	Mu	22.18	0.02	4616.72	15071.16	61332.82	57.26	67344.38	189219.70	21623.58	6.66	821.34	0.22	10.48	143.54	1.06	0.38	4.74	3.00	0.52
Sauvignon Blanc	Mu	9.66	0.74	5108.22	7214.10	56646.00	<0.001	71904.02	198556.40	30771.62	3.62	942.04	0.62	7.92	632.44	0.44	0.56	4.30	1.00	<0.001
Sauvignon Blanc	Mu	13.44	1.48	5225.14	8882.58	44671.74	176.82	53648.16	209012.00	32956.62	3.66	1040.26	1.48	22.36	215.56	0.26	0.18	9.76	2.04	0.10
Riesling Italian	Mu	17.22	2.94	4161.04	8478.50	56510.30	<0.001	128907.70	273361.40	37623.04	6.22	911.46	<0.001	40.42	40.78	0.64	0.36	3.58	0.96	0.20
Sauvignon Blanc	Mu	2.38	0.74	6463.64	4745.04	54138.24	13.08	40634.92	221838.50	45238.76	3.80	612.08	0.04	100.00	810.70	<0.001	0.12	10.10	0.70	0.52
Sauvignon	Mu	0.64	0.74	2822.80	3605.82	39961.84	<0.001	109704.10	310043.60	50328.94	1.72	361.56	0.08	24.24	406.00	0.20	0.16	7.46	0.16	0.84
Riesling Italian	O	10.54	0.74	4060.84	8976.06	64051.36	128.30	89434.02	215775.00	39865.44	8.62	938.90	<0.001	37.52	20.42	0.34	0.24	4.80	3.20	0.30
Chardonnay	O	9.08	<0.001	9120.38	5492.58	54533.40	67.38	95562.64	265486.5	54825.06	7.32	624.44	0.42	107.08	125.48	0.74	1.48	14.74	2.10	0.42
Sauvignon Blanc	O	5.00	0.02	5795.44	5460.40	53304.28	148.98	61770.18	221251.1	25173.24	5.46	445.00	0.72	25.00	343.22	0.24	0.48	4.10	3.38	0.30
Riesling Italian	O	8.50	0.02	2663.00	5417.82	56411.86	0.22	93927.36	209704.4	31886.86	6.08	660.84	0.04	18.18	65.22	0.70	0.08	5.12	0.86	0.30
Chardonnay	O	8.78	1.48	3776.96	7151.30	55328.72	<0.001	65486.6	227405.6	28097.46	9.06	506.32	<0.001	<0.001	214.54	0.46	0.24	5.06	1.74	0.20

Sauvignon Blanc	O	0.06	0.02	7571.08	12019.78	51416.64	88.9	70472.76	289726.80	50408.06	4.32	381.50	<0.001	0.56	401.8	0.44	0.26	5.86	0.38	0.2
Riesling Italian	O	5.88	0.02	5081.98	5381.34	49784.86	<0.001	153064.80	373798.50	40036.18	4.68	522.26	<0.001	18.18	371.94	1.06	0.38	4.22	0.46	<0.001
Chardonnay	O	0.94	0.02	5022.32	10889.14	54372.12	25.94	123819.10	273494.30	49531.26	3.14	690.04	0.04	8.60	131.86	0.50	0.26	4.30	1.20	0.20
Sauvignon Blanc	O	2.96	0.02	5532.96	5904.76	61005.8	55.64	39238.96	226440.4	48438.16	5.12	414.08	0.72	17.38	349.12	<0.001	0.34	3.38	0.64	0.52
Riesling Italian	O	6.16	0.02	2443.56	5200.86	49648.04	285.38	56373.10	138459.20	34231.28	3.84	327.46	<0.001	5.12	262.24	0.10	0.24	6.24	0.70	0.72
Chardonnay	O	8.50	0.02	5528.18	8685.86	56431.04	165.14	61207.18	242155.40	40741.96	5.08	421.98	0.36	9.24	374.50	0.40	0.80	4.64	0.58	0.42
Pinot Gris	O	9.96	0.74	2696.40	6381.48	60010.26	<0.001	95604.24	214523.10	35269.08	4.54	641.42	0.06	23.30	168.90	0.14	0.24	14.38	0.94	0.62
Chardonnay	O	2.38	2.20	2844.28	3211.06	52413.02	<0.001	46633.62	245729.20	48779.74	4.76	477.04	<0.001	128.48	183.60	0.04	0.24	11.94	0.50	0.10
Riesling Italian	O	15.48	0.02	1654.16	3889.98	55610.70	<0.001	72224.96	198003.90	48927.78	4.48	281.56	0.28	42.72	49.36	0.66	0.44	18.02	0.48	0.10
Sauvignon Blanc	O	1.80	0.02	5015.18	5560.08	55641.28	49.06	52345.58	182526.70	43064.28	4.08	319.08	0.66	<0.001	269.44	<0.001	0.08	2.92	1.08	0.10
Riesling Italian	O	8.500	<0.001	2932.52	12085.18	52744.50	199.28	111465.70	144236.00	33468.76	7.04	468.54	0.68	66.78	927.10	0.14	0.12	4.38	0.06	0.20
Chardonnay	O	6.76	1.48	4545.14	6302.48	55001.98	221.02	89523.30	199843.20	44874.44	5.06	572.52	0.88	31.34	766.22	0.14	0.24	3.72	1.94	<0.001
Sauvignon Blanc	O	14.02	0.74	7069.84	11277.8	47307.14	<0.001	132527.00	395898.80	54188.78	9.62	385.50	1.36	62.22	189.12	0.76	0.46	6.66	4.62	<0.001
Riesling Italian	M	15.48	0.02	4430.62	5921.22	67676.72	540.02	67825.50	251177.30	54143.22	7.36	632.96	1.48	35.82	900.76	0.60	0.56	7.70	0.64	0.20
Sauvignon Blanc	M	13.44	<0.001	7864.68	19440.26	54921.88	<0.001	83002.84	463549.20	21907.98	7.48	703.88	0.88	36.62	146.86	0.54	1.22	7.48	0.84	0.10
Riesling Italian	M	75.14	1.48	6971.98	5324.66	74393.56	551.46	38341.56	154663.70	41743.72	6.26	563.34	1.88	<0.001	97.24	0.30	0.48	3.76	0.30	1.46
Sauvignon Blanc	M	28.58	0.74	7162.92	3416.64	61881.20	936.24	96717.58	197850.00	23318.72	7.64	497.70	7.60	0.22	305.98	0.86	0.34	5.18	1.42	0.62
Chardonnay	M	33.82	0.02	7750.10	5619.46	51515.10	941.44	102560.40	285306.70	20474.56	5.76	496.88	4.22	33.00	292.82	1.10	0.70	5.08	2.14	0.84
Pinot Gris	M	27.42	0.02	8203.66	3719.30	80258.88	524.00	66869.32	214404.20	33058.02	4.94	994.28	4.68	65.24	198.30	0.84	0.48	3.02	2.60	0.84
Riesling Italian	M	21.60	5.88	5151.18	34053.46	63701.26	1217.76	93874.26	123688.40	48157.36	4.82	1569.36	5.06	5.42	1116.94	0.76	0.30	22.16	0.56	<0.001
Sauvignon Blanc	M	10.82	<0.001	4769.42	9021.02	52828.48	<0.001	148327.20	352509.60	28689.84	3.26	609.56	<0.001	2.98	143.08	0.96	0.74	3.94	0.02	0.10
Pinot Gris	M	<0.001	0.02	3113.80	3333.68	48500.66	76.34	81329.18	151621.40	41504.66	2.76	499.94	0.08	172.42	363.92	0.16	<0.001	27.36	0.84	0.30
Riesling Italian	M	7.34	2.94	3717.32	6346.74	56331.22	148.18	58662.92	184464.00	52161.70	3.14	1211.26	4.52	121.32	257.94	0.24	0.36	23.80	1.02	0.84
Sauvignon Blanc	M	8.78	0.02	5017.56	7361.90	53676.08	30.18	114028.00	299371.20	26663.76	3.38	633.78	<0.001	<0.001	221.22	0.64	0.84	3.16	0.40	0.30
Riesling Italian	M	16.64	0.02	6139.08	4594.62	65253.24	<0.001	81229.70	267500.70	32022.8	5.28	956.16	0.02	21.68	125.04	0.30	0.48	4.74	1.52	<0.001
Sauvignon Blanc	M	9.66	0.74	6394.42	12615.84	57807.84	25.14	108546.90	369427.50	25730.76	11.06	669.48	0.08	26.96	111.08	0.66	0.90	13.20	0.94	0.42
Riesling Italian	M	15.48	0.02	4048.92	6409.60	53197.22	334.14	98692.60	184387.10	42586.14	4.64	871.18	2.78	12.06	246.52	0.70	0.30	6.62	3.22	0.20
Sauvignon Blanc	M	14.32	<0.001	5110.62	6748.66	49116.62	458.70	113697.40	225440.30	52958.86	5.44	992.68	3.88	4.56	219.58	1.48	0.46	4.54	0.46	2.18
Chardonnay	M	4.72	0.02	6019.76	6612.22	54482.24	476.82	143806.60	225321.40	33639.46	4.94	1153.20	5.72	15.94	278.94	0.80	0.42	5.30	1.34	1.26
Pinot Gris	M	11.12	0.02	5354.00	6354.88	68368.60	362.02	119169.90	214348.20	32478.64	3.88	1221.24	2.66	24.92	357.40	0.54	0.24	3.32	0.14	1.04
Sauvignon Blanc	M	22.76	0.02	6296.58	22482.74	67612.22	1857.86	109976.60	231196.20	27847.14	5.04	1024.24	6.58	101.32	534.76	0.90	0.30	4.46	0.64	0.84
Pinot Gris	M	9.96	1.48	6248.86	5942.46	62143.98	912.94	109806.00	272333.40	25821.78	4.66	1158.28	5.20	17.42	255.1	0.94	0.26	6.48	1.96	0.72

As	Rb	Sr	Pd	Ag	In	Sb	Cs	Ba	Ce	Au
1.18	1595.48	122.46	10.68	1.80	0.32	0.28	4.38	49.96	3.88	0.18
2.02	2028.16	143.44	7.50	0.46	0.44	0.32	3.70	20.52	0.12	0.42
2.68	1351.00	129.30	8.12	0.38	1.16	0.36	5.64	56.18	1.82	0.46
0.50	2243.74	220.48	6.18	0.38	0.16	0.52	8.92	54.14	2.08	0.26
<0.001	310.74	246.16	5.72	0.42	0.04	0.06	0.70	51.18	0.88	0.28
<0.001	495.14	259.20	5.62	0.46	0.06	0.10	1.68	65.36	0.90	0.28
4.04	3025.54	202.32	3.56	0.54	<0.001	0.02	9.38	39.28	0.74	0.42
<0.001	896.54	134.80	2.12	0.20	0.08	0.20	3.60	78.14	18.94	0.12
1.52	1279.80	125.78	4.06	<0.001	<0.001	0.38	3.94	49.70	0.68	0.08
0.34	509.74	151.14	1.36	0.20	0.14	0.24	1.56	41.22	1.66	0.42
0.16	677.52	218.38	<0.001	<0.001	<0.001	0.12	2.22	65.88	1.30	0.42
0.34	715.46	118.62	0.04	<0.001	0.84	0.08	2.08	34.44	0.68	0.18
0.68	1333.32	201.52	0.42	0.06	<0.001	0.14	4.18	148.38	2.10	0.22
1.34	1331.02	431.50	0.50	0.12	0.02	0.32	3.66	174.98	2.88	0.24
0.68	2340.86	304.50	0.18	0.28	<0.001	0.36	2.54	146.98	0.86	0.22
1	1589.90	334.48	0.84	0.28	0.14	0.18	3.72	49.40	0.48	1.00
<0.001	1043.12	184.98	5.36	0.22	0.14	0.14	2.04	77.04	3.60	0.28
0.34	1387.14	158.08	2.18	0.10	0.16	0.32	2.38	50.56	0.42	0.12
1	587.68	151.20	3.12	0.34	0.04	0.14	1.72	60.80	0.02	0.38
<0.001	895.16	268.68	4.30	0.74	0.02	0.20	2.60	170.66	32.18	0.32
0.84	697.48	529.12	3.60	0.38	0.18	0.50	1.48	118.54	0.60	0.18
0.84	881.80	1035.94	10.68	0.26	0.10	0.54	3.32	135.52	1.14	0.18
1.18	1095.42	393.44	3.86	0.04	0.46	0.30	6.30	101.68	0.24	0.12
0.34	1176.32	332.92	0.26	0.04	0.14	0.28	5.54	60.64	1.08	0.22
2.18	737.80	353.58	14.44	0.42	0.02	0.22	3.52	123.40	0.24	0.22
1	644.38	406.46	<0.001	0.04	0.06	0.32	1.98	93.56	0.68	0.66
0.5	538.24	292.56	1.56	0.04	0.08	0.28	2.48	87.06	0.56	0.42
0.84	1376.48	185.98	2.74	<0.001	0.06	<0.001	2.56	56.86	1.14	0.58
3.02	984.26	303.78	4.56	0.48	0.62	0.44	4.60	79.70	0.28	0.32
<0.001	2157.36	200.58	3.38	<0.001	0.32	0.22	7.78	86.84	2.20	<0.001
1.84	1194.50	148.36	14.38	0.58	0.08	0.24	5.52	86.70	0.24	0.96
0.68	734.02	309.20	8.68	0.98	0.30	0.44	4.14	67.32	0.64	0.42
2.68	847.24	174.02	4.44	<0.001	0.38	0.08	6.80	84.34	94.00	0.02
<0.001	1353.54	143.52	1.06	0.16	0.08	0.32	3.72	83.14	0.34	0.08

1.84	977.76	209.86	0.18	0.08	<0.001	0.28	2.62	61.40	<0.001	0.14
0.50	1460.42	205.42	1.36	0.38	0.06	0.08	6.90	76.36	0.84	0.22
0.50	1288.36	182.92	4.94	0.42	0.14	0.24	4.18	30.54	1.38	0.32
<0.001	607.64	136.96	1.86	0.66	0.02	0.16	4.30	197.76	23.14	0.56
1.00	1674.84	258.18	2.86	0.26	<0.001	0.14	10.22	95.68	0.48	0.42
0.16	1123.40	318.08	1.74	0.14	<0.001	0.16	4.16	67.40	0.28	0.38
0.68	1613.30	311.04	1.06	0.04	0.14	0.14	5.54	62.20	1.64	0.02
<0.001	1215.40	214.38	2.92	0.22	0.12	<0.001	7.44	71.46	1.22	0.02
<0.001	1028.22	121.76	0.70	0.12	0.20	0.1	2.26	45.30	0.62	0.12
<0.001	590.50	267.74	0.36	<0.001	0.16	0.02	2.02	69.14	0.62	0.08
<0.001	770.58	184.20	3.04	0.54	0.12	0.02	3.62	77.00	0.38	0.48
<0.001	1811.36	196.66	6.00	0.30	0.22	0.32	4.64	67.42	1.30	0.70
2.02	1228.70	248.26	8.38	0.70	0.34	0.24	2.80	66.28	0.56	0.32
0.50	2448.06	399.48	7.26	0.12	0.08	0.24	9.90	71.74	0.04	0.08
1.68	1130.62	576.58	5.94	0.12	<0.001	0.28	4.00	89.52	17.74	0.28
3.20	1396.04	459.44	8.32	0.6	<0.001	0.32	3.84	111.84	4.46	0.06
2.86	1714.22	414.82	8.38	0.12	<0.001	0.18	4.80	143.58	2.44	0.16
1.34	1533.10	953.38	7.18	0.76	0.64	0.20	4.60	59.10	1.18	0.56
1.18	602.34	594.72	5.74	0.54	0.10	0.22	3.22	54.96	4.34	0.26
0.68	1228.22	208.24	0.92	0.06	<0.001	0.18	4.10	220.18	2.22	0.38
0.68	593.90	153.92	4.68	<0.001	0.02	0.24	1.86	43.62	2.22	0.14
0.34	1023.20	288.98	3.9	0.22	0.10	0.22	3.14	64.22	0.52	0.32
<0.001	1876.08	238.66	1.36	0.68	<0.001	0.40	3.82	83.00	<0.001	0.32
0.68	1394.48	438.98	3.60	0.18	0.06	0.30	4.50	124.64	<0.001	0.08
<0.001	1893.00	249.46	7.30	1.76	<0.001	0.36	7.04	101.28	0.48	0.08
1.84	909.16	303.36	2.42	0.08	0.16	0.22	5.14	62.90	2.60	<0.001
1.68	1324.82	334.92	2.42	0.08	0.04	0.18	2.56	96.42	24.92	0.08
2.18	1513.44	375.40	<0.001	0.20	0.02	0.06	3.52	85.32	0.50	0.08
1.34	1331.24	675.02	<0.001	0.08	0.08	0.14	2.72	76.16	2.12	0.22
2.36	1493.98	587.24	3.56	0.14	0.06	0.18	6.60	116.98	17.18	0.28
2.36	1560.84	648.30	11.56	0.12	0.44	0.46	4.78	147.12	3.16	0.38
